# The Echobot: An automated system for stimulus presentation in studies of human echolocation

**DOI:** 10.1371/journal.pone.0223327

**Published:** 2019-10-04

**Authors:** Carlos Tirado, Peter Lundén, Mats E. Nilsson

**Affiliations:** 1 Gösta Ekman Laboratory, Department of Psychology, Stockholm University, Stockholm, Sweden; 2 Research Institute of Sweden, Borås, Sweden; University College London, UNITED KINGDOM

## Abstract

Echolocation is the detection and localization of objects by listening to the sounds they reflect. Early studies of human echolocation used real objects that the experimental leader positioned manually before each experimental trial. The advantage of this procedure is the use of realistic stimuli; the disadvantage is that manually shifting stimuli between trials is very time consuming making it difficult to use psychophysical methods based on the presentation of hundreds of stimuli. The present study tested a new automated system for stimulus presentation, the Echobot, that overcomes this disadvantage. We tested 15 sighted participants with no prior experience of echolocation on their ability to detect the reflection of a loudspeaker-generated click from a 50 cm circular aluminum disk. The results showed that most participants were able to detect the sound reflections. Performance varied considerably, however, with mean individual thresholds of detection ranging from 1 to 3.2 m distance from the disk. Three participants in the loudspeaker experiment also tested using self-generated vocalization. One participant performed better using vocalization and one much worse than in the loudspeaker experiment, illustrating that performance in echolocation experiments using vocalizations not only measures the ability to detect sound reflections, but also the ability to produce efficient echolocation signals. Overall, the present experiments show that the Echobot may be a useful tool in research on human echolocation.

## Introduction

Many blind people have learned to echolocate, that is, to detect and localize objects by listening to the sounds they reflect. Human echolocation has been studied since the 1940s using a variety of methods and stimuli [1–3]. Several early studies used real objects that the experimental leader positioned manually before each experimental trial [4–7]. The advantage of this type of experiment is that the stimuli have high ecological validity as participants are engaged in detecting or locating real sound reflections from real objects. The disadvantage is that manually shifting stimuli between trials is so time consuming that it makes it difficult to use psychophysical methods based on the presentation of several hundred stimuli. Therefore, many previous studies involving manually shifting stimuli typically involved no more than 100 trials per test occasion and participant [4, 5, 6, 8, 9]. This limitation to the number of test trials is overcome in earphone studies using prerecorded [10–12] or simulated [13–15] sounds that can be reproduced, manipulated, or generated in real time. However, the price of this is that the ecological validity may be questioned, because recorded or simulated sounds may not include all the relevant acoustic information available to a listener in real life. The present study tested a new automated system for stimulus presentation, the Echobot. It combines the use of ecologically valid stimuli with the use of rigorous psychophysical methods.

**Fig 1:**
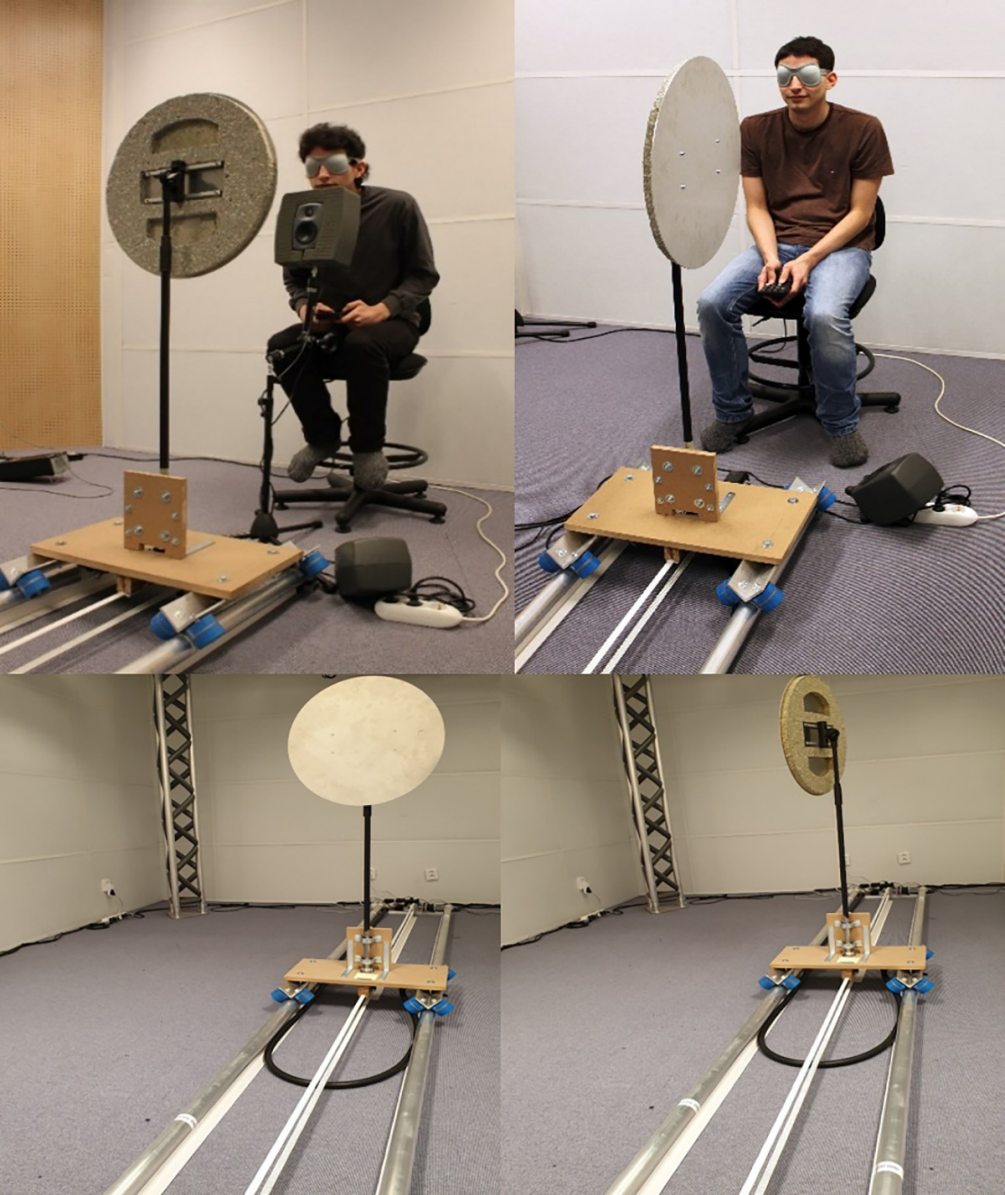
The Echobot. In a reflecting position (upper left picture) and in a non-reflecting position (upper right picture). The blindfolded participant responded using a wireless keyboard. The loudspeaker in front of the participant, covered with a sound absorbing material, generated the echolocation click in the loudspeaker experiment, and worked as a chin rest. In the vocalization experiment, the participant generated their own signals (upper right picture). In both experiments, the loudspeaker on the floor played a masking sound while the Echobot was moving and provided auditory feedback (“right” or “wrong”) after the participant had responded. The Echobot moved along a 4 m long rail. In the present setup, it could stop at distance between 0.7 and 3.9 m from the participant. After it stopped, the disk was either rotated to a reflecting position (lower left picture) or non-reflecting position (lower right picture). The individual in these pictures, who is one of the authors, has given written informed consent (as outlined in PLOS consent form) to publish these images.

**Fig 2:**
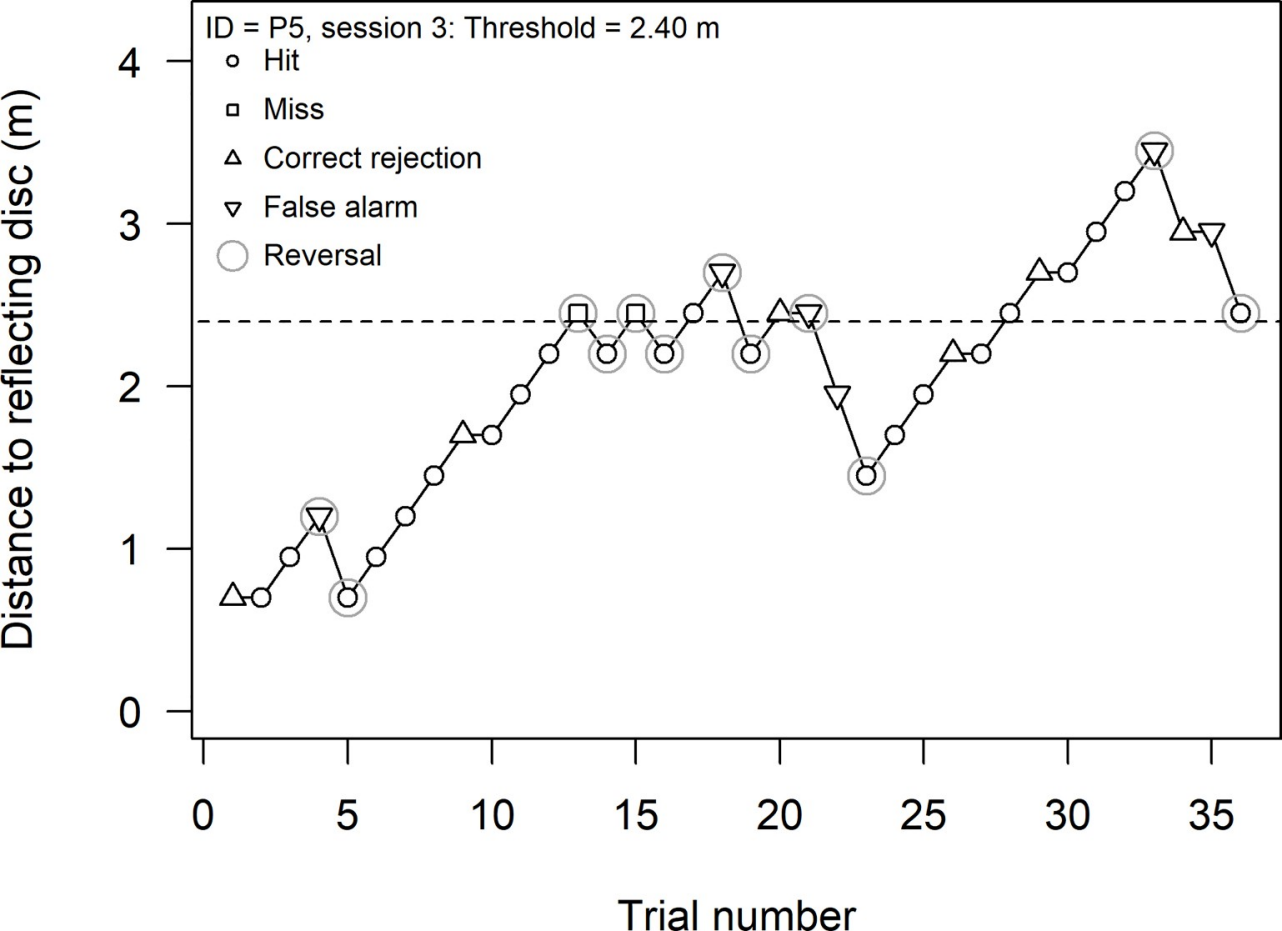
Example of one adaptive staircase. The staircase rule followed the single-interval adjustment matrix (SIAM [22]), which is an adaptive procedure for yes–no tasks targeting a threshold corresponding to a performance of d′ = 1.0 (i.e., 69% correct for an unbiased observer). The figure shows the staircase from one of participant P5’s sessions. The threshold estimate, 2.40 m (dashed line), was calculated as the mean of the 10 last reversals (reversals are circled). Hits are shown with circles, misses with squares, correct rejections with triangles, and false alarms with inverted triangles.

We built the Echobot to automatize stimulus presentation in experiments on human echolocation (Fig 1 and film [16]). The Echobot may be programmed to change the distance and the position of its reflecting object according to an experimental protocol such as following various rules of adaptive staircase methods (see Fig 2) often used in psychophysics to obtain threshold estimates [17]. If the object is a thin disk, as in the present experiments, the disk can be made virtually non-reflective by rotating it 90° so the sound-reflecting area—the disk’s edge—is too small to reflect audible sound. Thus, the disk can be positioned in a reflecting or non-reflecting mode, and a listener’s ability to hear the difference can be used as a measure of his or her ability to echolocate.

Below we present the results of the first experiments using the Echobot. The main purpose was to demonstrate the usefulness of the Echobot for acoustic measurement of stimuli and for conducting echolocation experiments. In addition to testing the Echobot, the experiments had the specific purpose of exploring at what distances sighted naive listeners would be able to detect reflections from a disk. Previous research using real objects or recordings of reflections from real objects suggest that sighted people with no experience of echolocation may detect a medium-sized object up to about 1 to 3 m (with real or simulated sounds) [1, 10, 13, 18]. Here we tried to verify these findings, using an adaptive staircase method that adjusted the distance to the reflecting disk according to the participant’s performance on previous trials. Whether the disk would be in a reflecting or non-reflecting position was decided randomly for each trial.

In the first experiment, a loudspeaker generated the echo signal. Previous research suggests that sighted people with no experience of echolocation may perform better with loudspeaker-generated clicks than if they generate their own vocal sounds [19]. It is difficult to generate mouth clicks that are loud enough to be useful for echolocation, so it may require training to improve a participant’s ability to produce the click. Participants also need perceptual training to learn to hear the relevant information in reflected sound. In a vocalization experiment, we asked three participants from the first experiment to test again with the Echobot over 6 days, but now using self-generated vocalizations rather than loudspeaker-generated clicks. The purpose was to see whether they would perform better or worse than in the previous experiment and if their performance would improve over the 6 days of testing.

## Method

### Materials

In the present setup, the Echobot could move back and forth along a 4 m-long rail (Fig 1 and film [16]). The rail consisted of two parallel aluminum tubes with a 50 mm diameter. The rails were attached to a beam on each end with a half coupler (a type of clamp used in the lighting industry), which facilitated simple dismounting from the rails. The target object was a circular aluminum disk with a 0.5 m diameter and 0.4 cm thick that could be turned 360° around its own vertical axis. The disk was mounted on an adjustable-height stand mounted on a platform. The platform rolled on eight long-board wheels that were mounted in pairs with the axis of the wheels tilted ±45° relative the horizontal plane to keep the platform in place on the rails. Two stepper motors drove the robot’s movement. The first motor was coupled to the pole that holds the screen and rotated the disk around its axis. The second motor drove the horizontal movement through a cog belt that was mounted between the supporting beams at each end of the rails. The spatial resolution of the Echobot was 0.5 cm as determined by the spacing of cogs on the cog belt. The motors were mounted with an elastic suspension and couplings to prevent the propagation of vibrations. Each motor was controlled by a Steprocker TMCM-1110 stepper motor controller connected to a Raspberry Pi 3 computer. The Raspberry Pi controlled the two motor controllers and communicated wirelessly with a client program using Bluetooth. The user communicated with the robot through a client library in Python which handled the Bluetooth communication between the Echobot’s Raspberry Pi and the computer that run the experiment and collected the data. A loudspeaker generated a masking sound while the Echobot was moving. To maximize the masking ability of the noise, it was a mix of several recordings of the Echobot in motion and thus had the same spectral composition. The masking noise at the position of the listener’s ears had an A-weighted maximum sound pressure level (time-weighting fast) of about 64 dB(A). This completely masked the sound of the rotation of the Echobot’s disk, who at 1.5 m distance generated a maximum of about 28 dB(A). The Echobot moving along the rail from 1.5 to 2.0 m distance generated sounds with a maximum level of about 50 dB(A). This sound was not possible to differentiate from the masking noise.

**Fig 3:**
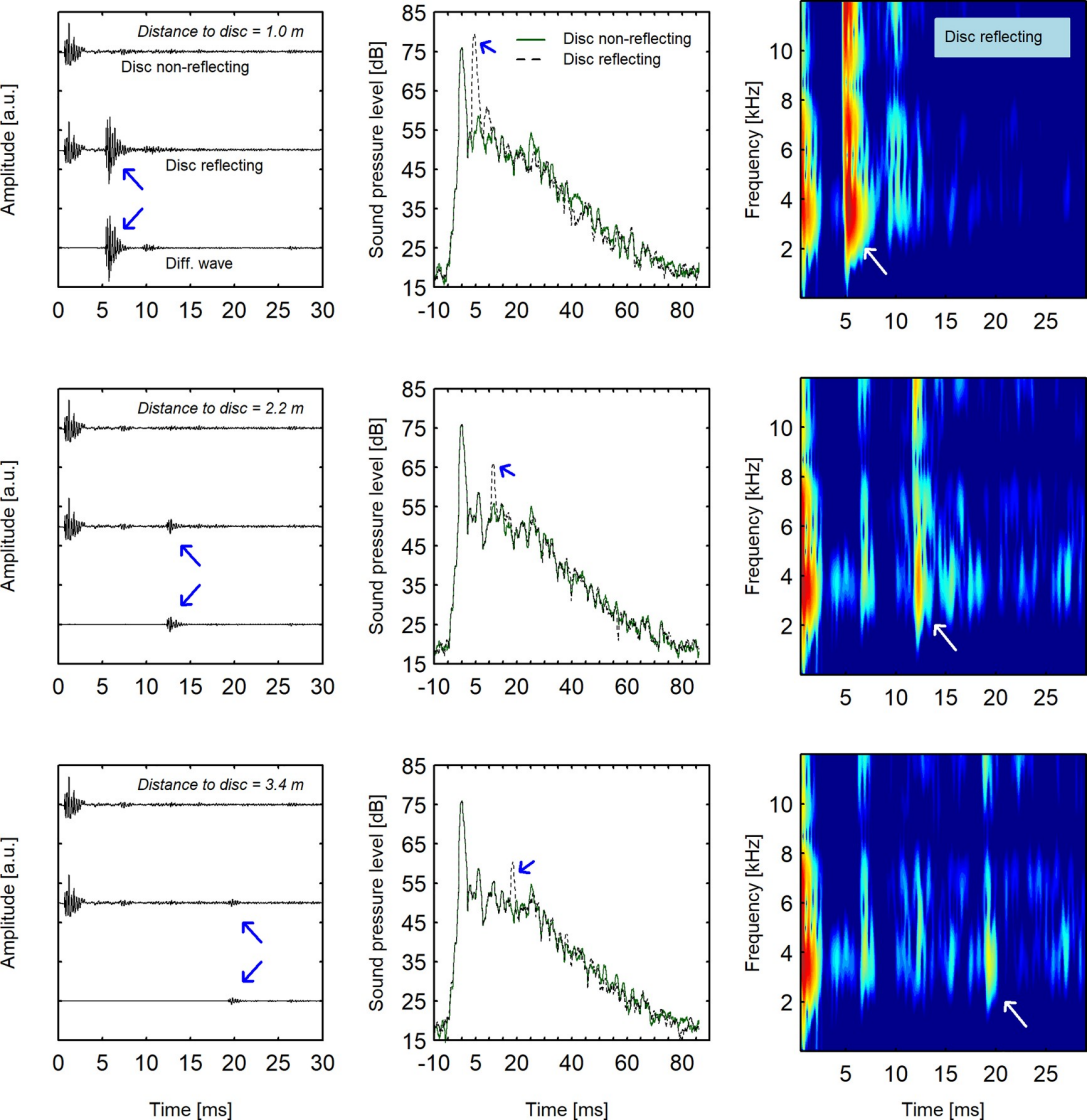
Acoustics of direct and reflected click. Visualization of signals recorded with the Echobot’s disk at 1.0 m, 2.2 m, and 3.4 m are shown in the upper, middle, and lower row of panels, respectively. Arrows indicate the position of the reflected click. *Left panels*: Amplitude (arbitrary unit) as a function of distance from the reflecting disk. In each panel, the three curves refer to recordings with the disk in non-reflecting position (upper), the disk in reflecting position (middle), and the wave difference obtained by subtracting reflecting from non-reflecting. *Middle panels*: Sound pressure level (1 ms running rectangular window) as a function of distance to the disk. The green solid curve refers to recordings with the disk in non-reflecting position; the dashed curve refers to recordings with the disk in reflecting position. Note that the time scale (x-axis) is longer than the time scale of the left and right panels. *Right panels*: Spectrograms (frequency vs. time, with color-coded amplitude) for recordings with the disk in reflecting position.

In the present study, the Echobot was placed in a soundproof listening laboratory (see Fig 1), with a low background level (< 20 dB[A]) and short reverberation time (< 0.1 between 0.25 and 8 kHz). The floor area was 5.3 × 4.0 m^2^ with a ceiling height of 3 m. With the exception of a loudspeaker rig intended for other types of experiments, the room was empty. The rig was built from 50 mm diameter aluminum pipes, largely covered with soft dampening material to eliminate resonance. Measurements showed that reflections from the rig were smaller than reflections from the disk (see Fig 3). It is important to note that the rig’s reflections were present in all trials, so they could not provide information about the disk’s position.

### Echolocation signals

In the loudspeaker experiment, the echo signal was taken from Thaler et al. [20], who simulated a click based on recordings of many mouth clicks generated by an experienced echolocator. The simulated click was 2 to 3 ms long and dominant frequencies were around 3 to 4 kHz (see [20] for a detailed acoustic characterization of the click called EE1). In the present experiment, the peak-to-peak sound pressure level of the click was set to 87 dB, as measured 1 m directly in front of the loudspeaker (Genelec Model 8010A). In the vocalization experiment, self-generated sounds were used. The participants were told that they could use any sound they liked, and they used either mouth clicks or a hissing noise of longer duration.

### Acoustic measurements

**Fig 4:**
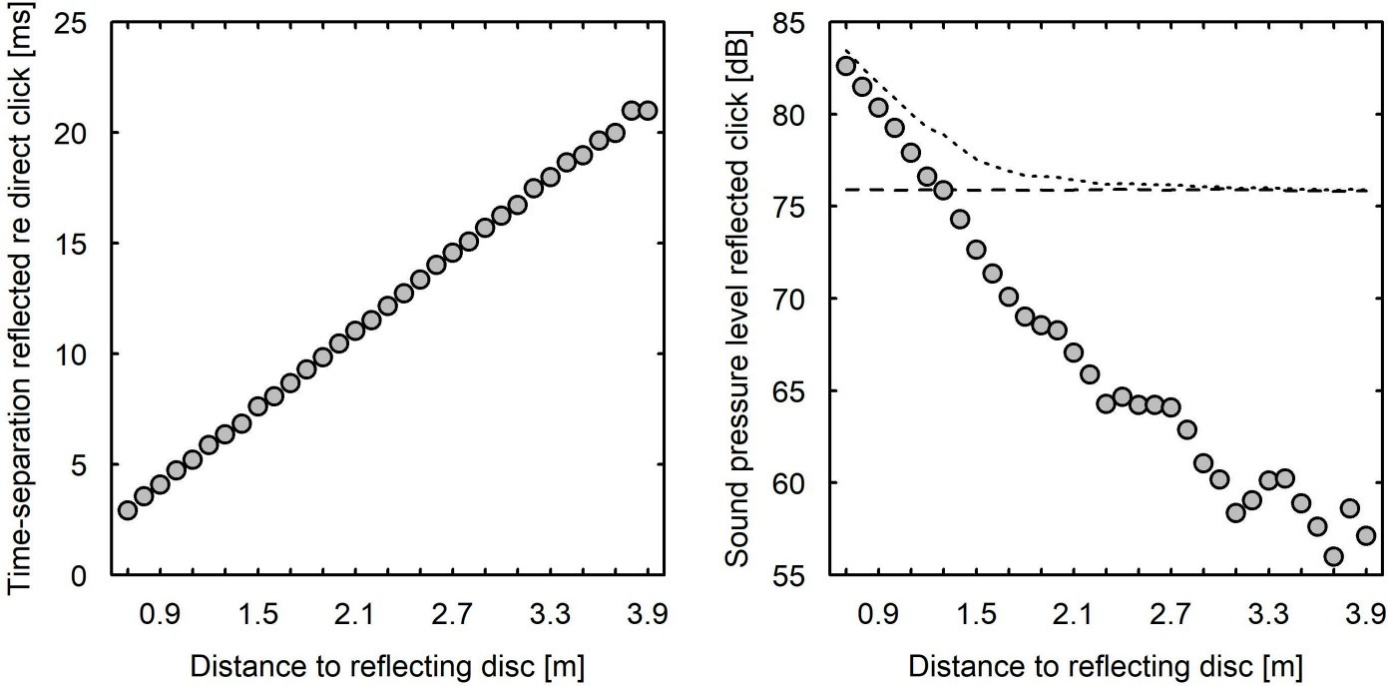
Time separation and SPL of the reflection as a function of distance to the disk. Time separation between direct and reflected click (left), and SPL (1 ms running rectangular window) of the reflected click as a function of distance from the reflecting disk in m (right).The horizontal dashed line in the right panel refers to the SPL of the direct click, and the dotted curve refers to the combined SPL of direct and reflected clicks.

Measurements were conducted in the Echobot using a measurement microphone (Brűel & Kjær, microphone type 4190, preamplifier type 2669, amplifier type 2690 NEXUS) connected to an external soundcard (RME Babyface Pro, 48 kHz sampling frequency, 24-bit depth) connected to a computer. The microphone was placed in the same location as the participant’s head in the experiments, just behind and above the loudspeaker that generated the echolocation click. Measurements were taken in steps of 0.1 m from 0.7 to 3.9 m distance to the reflecting disk. For each distance, 10 measurements of the echolocation signal were taken with the disk in reflecting mode and 10 measurements with the disk in non-reflecting mode. The 10 replicated measurements yielded very similar results, with standard deviations of less than 0.2 dB. The measurement results presented below are for individual measurements (Fig 3) or an average of the 10 measurements at each distance (Fig 4). From these measurements, sound pressure levels (SPLs) were calculated for running rectangular 1-ms windows with an overlap of 0.998 ms. The maximum of these SPLs in the initial part of the measurement (from 0–3 ms) referred to the direct sound and the maximum of a subsequent part (from 3–30 ms) referred to the reflected sound. The time separation between these maxima was used to calculate the inter-click interval (ICI), the time between the direct and the reflected sounds. All the statistical analyses were conducted using the statistical software R [21].

### Loudspeaker experiment

The participants were seated in the Echobot (Fig 1) and responded using a wireless keyboard connected to the computer controlling the Echobot. The participants were blindfolded during testing to eliminate visual cues, and a masking sound was played while the Echobot was moved to eliminate auditory cues from its movements. The time it took to move the Echobot varied from trial to trial depending on the staircase rule (see below), but once the wagon reached its position, the time to rotate the disk to a reflecting or non-reflecting position was always the same, so timing could not provide a cue to the correct answer. Once the Echobot came to a still, the masking sound ended and the loudspeaker generated the echo signal. The participant then pressed one of two keys on the keyboard corresponding to the responses “Yes, disk reflected” or “No, disk did not reflect.” Before the loudspeaker experiment, a training session was conducted, first without the blindfold to illustrate the difference between the reflecting and non-reflecting positions of the disk, and then with the blindfold at the closest distance to acquaint the participant with the task. The loudspeaker experiment started when the participant could reliably answer correctly at the closest distance or after about 20 trials (two listeners found the task difficult even at the closest distance of 0.7 m).

Each participant completed 12 sessions of one staircase test yielding one single-session threshold (see Fig 2). The staircase rule followed the single-interval adjustment matrix (SIAM, [22]), which is an adaptive procedure for yes–no tasks targeting a threshold corresponding to a performance of d′ = 1.0 (i.e., 69% correct for an unbiased observer). The participant’s answers were classified as (1) “Hit”, for a “Yes” when the object was in reflecting mode, (2) “Correct rejection”, for a “No” when the object was in non-reflecting mode, (3) “Miss”, for a “No” when the object was in reflecting mode, and (4) “False alarm” for a “Yes” when the object was in non-reflecting mode. Each type of answer affected the distance to the disk in the next trial as follows: (1) “Hit” increased the distance 25 cm, (2) “Correct rejection” did not affect the distance (disk remained where it was), (3) “Miss” decreased the distance 25 cm, and (4) “False alarm” decreased the distance 50 cm. The adaptive staircase continued until 12 reversals were reached, and the single-session threshold was defined as the average of the last 10 reversals (see Fig 2 for an example staircase). Over the 12 sessions, the participants completed between 421 and 773 trials (mean = 521), and each session consisted of 22 to 81 trials (mean = 43), depending on the performance of the listener. The median time for a trial was 9 s (95% of the trials were faster than 11 s), including time for the Echobot to move, the click to be presented (about 1 s), response time of participant, and time (about 1 s) for providing auditory feedback. There were breaks after sessions 3, 6, and 9 and the experiment lasted for 1.5 to 2 hours.

### Vocalization experiment

Three participants from the loudspeaker experiment also participated in a follow-up experiment in which they were tested for 6 days, 12 sessions per day. The procedure was the same as in the loudspeaker experiment, except that the participants used self-generated vocalizations and the loudspeaker was removed (see upper right photo, Fig 1). They were instructed to use any sound they could produce with their vocal organs and to repeat it as many times as they liked before responding. The participants were well aware of the fact that many expert echolocaters use mouth clicks and this was the type of sound that was used the most. The participants completed between 391 and 557 trials (mean = 477) per day, and each session consisted of 23 to 66 trials (mean = 40). The median time for a trial was 10 s (95% of the trials were faster than 23 s), including time for the Echobot to move, time for the participant to generate a vocalization (they could use as many sounds they liked) and give a response, and time (about 1 s) for providing auditory feedback.

### Participants

Fifteen participants were tested (mean age = 27 years) in the loudspeaker experiment, three of whom also participated in the vocalization experiment. The participants were all students at the Department of Psychology, Stockholm University, and they all provided informed consent to participate. The participants were sighted and wore a blindfold during the experiment. Two (participants P4 and P5) had previously participated in echolocation experiments. The participants’ hearing status was tested using an audiometer (Interacoustic Diagnostic Audiometer, model AD226). Pure-tone thresholds were measured with the Hughson Westlake method, testing each ear for the frequencies 0.5, 1, 2, 3, 4, and 6 kHz. The results showed that all participants had normal hearing, defined as ≤ 20 dB hearing level in the best ear of the tested frequencies. The raw data and scripts for every participant and figure can be found in Figshare [23]. The Regional Ethics Review Board in Stockholm approved the study. Approval number: 2017/170-31/1. Participants gave oral consent (which is approved by the Regional Ethics Review Board in Stockholm) and their data were coded and analyzed anonymously.

## Results

### Acoustic measurements

Fig 3 shows the acoustic measurements of clicks with the disk in a reflecting or non-reflecting position. The upper, middle, and lower row of panels show recordings at distances from the disk of 1.0, 2.2, and 3.4 m, respectively. The left panel shows time histories of the signal with the disk in non-reflecting (upper graph) or reflecting (middle graph) position; the middle panel shows 1-ms SPLs as a function of time, and the right panel shows a spectrogram of the signal with the disk in reflecting position.

As expected, and clearly seen in Fig 3, the amplitude of the reflected click decreased with distance whereas the time interval between direct and reflected clicks increased. Note that the amplitude of the reflected click was greater than that of the direct click in the upper panel (distance = 1 m). This is due to the directivity of the sound source relative to the receiver. The loudspeaker radiates less energy backward than forward, especially for high-frequency sounds, such as our clicks with dominant frequencies around 3 to 4 kHz (see spectrograms in right-hand panels). The receiver (microphone or ears of an echolocator in the experiment) was located behind the loudspeaker, so less sound would reach the receiver than would be transmitted forward. In contrast, the reflecting disk was located directly in front of the receiver and reflected most of the sound back to the receiver (the dominant frequencies of the click corresponded to wavelengths < 0.12 m, which would be reflected by the 0.5 m disk). The plots of 1-ms SPLs as a function of time (middle panels of Fig 3) visualize the decay of the signal due to sound reflections in the room (the reverberation time in the room was about 0.07 s for frequencies >2 kHz). The reflected sound is clearly visible as a bump in the decay pattern at a time related to the distance from the disk.

Fig 4 shows results for a series of measurements with the disk located between 0.7 and 3.9 m in front of the receiver, in either reflecting or non-reflecting position. The left panel shows the ICI between the direct and reflected sounds as a function of distance from the reflecting disk. As expected, this relationship was linear, with an ICI increase of about 5.8 ms per meter increase of the distance from the disk. Except for the closest distance of 0.7 m (ICI = 2.9 ms), the time separation was larger than the duration of the click (3 ms). The right panel of Fig 4 shows the maximum SPL (1 ms rectangular window) as a function of distance separately for the direct sound (horizontal dashed line) and the reflected sound (circles). The SPL of the direct sound was almost identical in all recordings, with a maximum of 76 dB. As pointed out above, the reflected sound may have a stronger amplitude than the direct sound due to the directivity of the sound source relative to the receiver. This was the case up to about 1.2 m distance, after which the reflected click had a lower level than the direct sound, down to a reflected-to-direct ratio of about −20 dB at the farthest distances. The level of the reflected sound decreased by roughly 11 dB per doubling of distance up to about 2 m, after which the relationship became less systematic, presumably because of reflections from walls and objects in the room. At the largest distance, 3.9 m, the reflected sound had an SPL of about 56 dB. This is clearly above the background level of the room (<20 dB). However, the short duration of the click (about 3 ms) may make it difficult to detect, as the detectability of sounds increases with duration [24]. To clarify this, we put a loudspeaker at 3.9 m distance and played the click at a level that generated 56 dB at the point of the receiver. This click was soft but still clearly audible.

### Loudspeaker experiment

**Fig 5:**
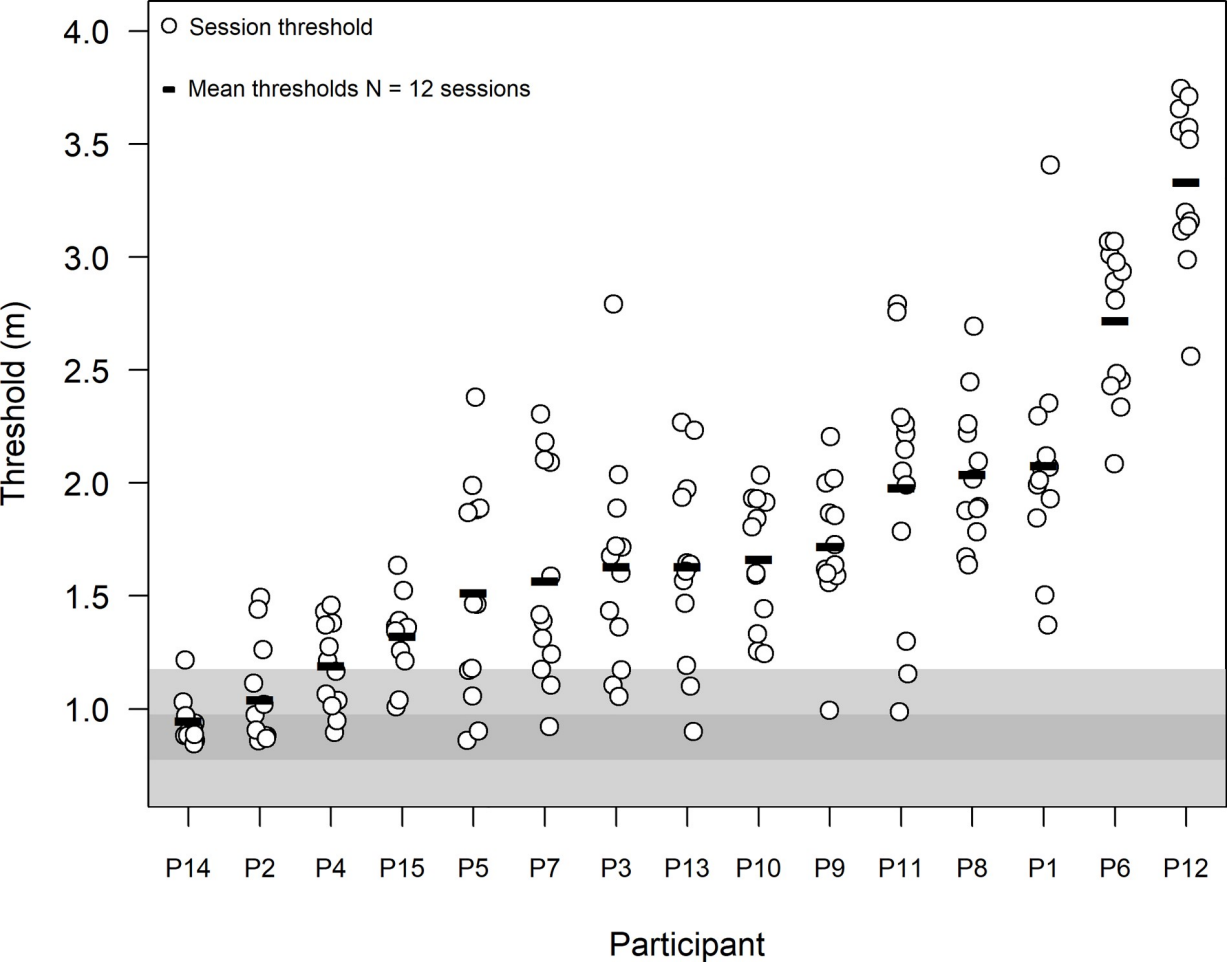
Loudspeaker experiment: Participant’s single-session and individual mean detection thresholds. Circles show individual threshold estimates in a gray scale, where the 1st to 4th session are light gray, the 5th to 8th session are medium gray, and the 9th to 12th session are dark gray. The black bar shows the mean threshold estimate over the 12 sessions. The individual results are displayed along the x-axis in order from the lowest to the highest performing participant. Individual threshold estimates from a random responder would fall in the light gray area 95% of the time and mean estimates of a random responder (12 sessions) would fall in the dark gray area 95% of the time.

Each participant performed 12 sessions in the echolocation experiment, corresponding to 12 staircases of the adaptive method (see Fig 2 for one staircase example). For each participant, the mean threshold estimate (thick lines in Fig 5) was defined as the mean of the 12 single-session threshold estimates (circles in Fig 5). We conducted simulations of a random responder by simply running the experimental script 10,000 times (with no sounds and Echobot turned off) with random answers. These simulations showed that the mean threshold estimate of an observer responding randomly would fall in the range of 0.88 to 0.98 m 95% of the time (dark gray region in Fig 5) with 95% of the single-session threshold estimates in the interval 0.8 to 1.2 m (light gray region in Fig 5).

There was considerable variation across individuals in mean threshold estimates (Table 1, Column 1). Two of the participants (P2 and P14) performed close to chance level; two (P4 and P15) performed better than chance with a mean threshold of 1.2 to 1.3 m; six (P3, P5, P7, P9, P10, P13) attained a distance of 1.5 to 1.7 m, and three (P1, P8, P11) performed around 2 m. P6 and P12 performed best, at 2.7 and 3.3 m respectively. Among the participants performing better than chance, the difference between the mean of the best (P12) and the worst (P4) performing participants was about 2 m.

**Table 1:** Individual data: Mean thresholds, correlation between single-session threshold and session number, and self-reported strategies.

Participant^a^	Mean threshold estimate^b^ [m]	Spearman’s rank-order correlation^c^	Self-reported strategy^d^
P14	0.95	−.47	Sharper sound when disk is reflecting
P2	1.0	.01	Different sound when disk is reflecting
P4	1.2 (1.5)	−.43	Sharper sound when disk is reflecting
P15	1.3	.38	Hear two sounds
P5	1.5 (2.3)	−.08	Sharper sound when disk is reflecting
P7	1.6	−.10	Sharper sound when disk is reflecting
P3	1.6	.24	Sharper sound when disk is reflecting
P13	1.6	.68	Sharper sound when disk is reflecting
P10	1.7	.35	Cannot explain
P9	1.7	.13	Hear two sounds
P11	2.0	.17	Hear two sounds
P8	2.0	.23	Cannot explain, it is a feeling
P1	2.1	.46	Cannot explain
P6	2.7 (1.1)	−.10	Hear two sounds
P12	3.3	.20	Hear two sounds

^a^ In order from lowest (worst) to highest (best) mean threshold.

^b^ Based on 12 single-session estimates from the loudspeaker experiment. Values in parentheses refer to mean threshold estimates from the vocalization experiment, based on 6 × 12 = 72 single-session estimates.

^c^ Calculated between the 12 single-session estimates and the order of the 12 sessions (1–12) in the loudspeaker experiment.

^d^ As reported after conducting the last session of the loudspeaker experiment.

To quantify training effects, we calculated the Spearman’s rank-order coefficient of correlation between single-session thresholds and session number separately for each participant (Table 1, Column 3). For most participants, these coefficients were small or negative, indicting lack of strong improvement during testing. Visual inspection of the individual data suggested trends of improvement only for 2 participants, P13 and P1, who also had the highest rank-order correlations, Spearman’s r = 0.68 and 0.45, respectively. Participant P13’s thresholds were around 1 m for the first three sessions, and then increased to about 1.5 to 2 m in the following sessions. Participant P1’s thresholds were around 1.5 m in the first two sessions, then increased to around 2 m in sessions 3 to 11, and increased markedly to 3.4 m in the last session. It is hard to tell whether this last-session jump in performance was due to luck, to a real improvement, or a combination of both.

After the last session, we asked participants to describe the strategy they used try to detect the reflected sound (Table 1, Column 4). These answers could be broadly classified into two groups. The first category included answers that described the sound as sharper when the disk was in a reflecting position than a non-reflecting position. These answers did not distinguish between the direct and reflected sounds and were presumably based on a perceptually fused perception of the two. The second category included answers that mentioned hearing two sounds when the disk was reflecting and only one when it was not, thus suggesting that the participant perceived the reflected sound as a separate event. There was a tendency for the best performing listeners to report the latter strategy.

### Vocalization experiment

**Fig 6:**
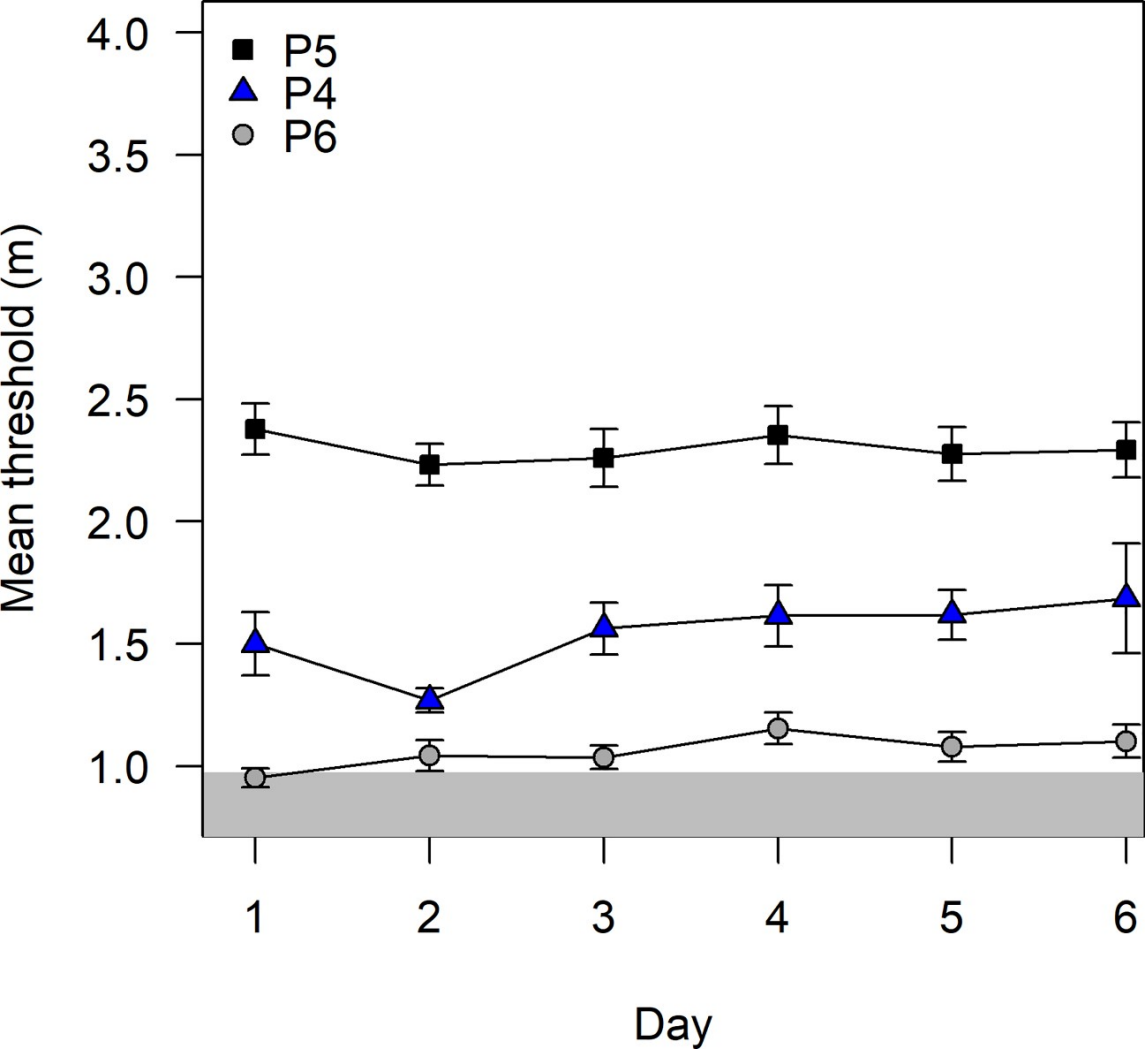
Vocalization experiment: Mean thresholds of each participant as a function of day. Individual mean thresholds (n = 12 sessions) in the vocalization experiment as a function of day of testing for participants P5 (black squares), P4 (blue triangles), and P6 (gray circles). Error bars show ±1 standard error of the mean. The mean threshold estimates of a random responder over 12 sessions would fall in the gray area 95% of the time.

Three of the participants in the loudspeaker experiment also volunteered to echolocate using self-generated sounds in the Echobot for 6 days of testing (12 sessions per day). Fig 6 shows mean thresholds per day (based on 12 sessions × 6 days) for each participant, with error bars denoting ± 1 standard error. The mean thresholds over all sessions and days were 1.5, 2.3, and 1.1 m, for participants P4, P5, and P6, respectively. These may be compared with the previous experiment in which mean thresholds for the same three participants were 1.2, 1.5, and 2.7 m, respectively. Thus, P4’s performance remained about the same, whereas P5 performed better and P6 much worse than in the previous experiment. All 3 participants complained that it was strenuous to repeatedly produce vocalization of sufficient intensity. P6 in particular found it difficult to produce a useful signal, which explains her close-to-chance performance in this experiment (her performance was second best in the previous experiment, Table 1). No participant improved substantially over time, as seen by the flat patterns of thresholds versus day (Fig 6).

## Discussion

These results demonstrate the usefulness of the Echobot for recording stimuli and running echolocation experiments. The results of the experiments showed that most participants were able to detect sounds reflected by the Echobot’s disk. However, performance varied considerably, with mean thresholds of detection ranging from 1 to 3.3 m distance from the disk. Three of the participants in the loudspeaker experiment also tested using self-generated sounds. One participant performed better and one much worse than in the loudspeaker experiment, illustrating that performance in echolocation experiments with self-generated sounds involves both ability to detect sound reflections and the ability to produce efficient echolocation signals.

Our study is the first to measure detection thresholds using both a real sound-reflecting object and an adaptive psychophysical method with an automated system. The results broadly agree with previous research using real objects, suggesting that sounds reflected by a medium-sized object can be detected by naive sighted listeners up to about 1 to 3 m [1, 10, 13, 18]. Our results add to this research by demonstrating large individual variability in performance. A few participants’ performances were close to chance, while the best participants could detect the disk up to distances over 3 m. The reason for this variability remains unclear, but it was interesting to note that most of the best performers indicated that their detection cue was hearing two separate sounds, whereas those who performed less well indicated that they listened to the loudness or sharpness of the sound. Perhaps manipulating these cues or the participants’ focus on them would be a good way to explore these individual differences.

Most participants had thresholds around 2 m, and the best had a threshold around 3.3 m. Why was the reflection not detected at farther distances? Acoustic measurements with the disk at 3.4 m (beyond the threshold of the best participant), showed that the 1-ms maximum SPL was about 60 dB by the time the reflection arrived at the participant’s ear, i.e., after around 20 ms. This SPL was clearly above the background level of the room (<20 dB), and it would be clearly above the auditory threshold if heard alone, as we verified in an informal listening test. It also seems unlikely that the direct click forward-masked the reflection. The click had a SPL of about 76 dB at 0 to 3 ms and 60 dB at 20 ms when the reflection arrived. This direct–reflected difference of −16 dB would be too high for complete forward masking to take place; a recent psychoacoustic study using short clicks found that a lag click following a lead click after 16 ms could be detected down to a lag–lead ratio of −40 dB or less [25].

The critical comparison, however, may not be with the background sound level of the room or the sound level of direct click, but with the sound level when the disk was in a non-reflecting position. The room was not anechoic, so reflections in the room could also have contributed to the SPL when the disk was non-reflective. With the disk at 3.4 m distance in a non-reflecting position, the SPL after 20 ms would be about 53 dB at the ear of the participant. This should be compared with 60 dB at the same time (i.e., when the reflection arrived) when the disk was in a reflecting position. This difference between reflecting and non-reflecting positions of the disk is seen as a bump in the decay pattern (see Fig 3, middle panels, bump indicated by an arrow). This bump may be difficult to detect as a separate event given that it appears somewhere within a complex decay pattern of about 70 ms. Our results indicate that this was indeed impossible at distances greater than about 3.2 m.

The results of the loudspeaker experiment suggested no substantial improvements over the 12 sessions. This is not surprising given that the test was done in a single day and improvement in echolocation would presumably require many days of training. The lack of substantial improvement in the vocalization experiment was more surprising, as the three participants conducted 12 sessions a day for more than 2600 trials in total per participant over the 6 days of testing. Previous studies have indeed found it possible for sighted people to learn to echolocate in various tasks using self-generated sounds (e.g., [10, 19, 26]). Our experiment involved self-generated vocalizations; therefore, a good performance would require both the ability to produce efficient echo signals and the ability to detect sound reflections. Apparently, the training time was not enough to improve both of these skills. All three participants complained that it was difficult and strenuous to produce a sound loud enough to be efficient for echolocation. This extra effort may have reduced the participants’ resources and thus ability to focus on sound reflections. Maybe it is necessary to specifically train the ability to produce efficient vocalizations, before aiming at an improvement in the ability to detect reflections (as in [27] In our study we did not attempt this, and that may explain the lack of sizeable training effects.

Thaler and Castillo-Serrano [19] compared the detection of reflections originating from either a loudspeaker or a mouth-generated click. Sighted participants new to echolocation generally did better when they used a loudspeaker than when they used mouth clicks, whereas 2 blind participants with experience in echolocation did equally well with mouth clicks and with the speakers. The equal performance of the blind participants supports the ecological validity of using loudspeaker-generated sounds instead of self-generated sounds in echolocation studies focusing on measuring auditory such as detection or localization of sound reflections. The obvious advantage of loudspeaker-generated sounds is that every participant is exposed to the same sound, so difference in performance may be attributed to their auditory abilities rather than to their abilities to produce efficient vocalizations. Thaler and Castillo-Serrano measured the mouth clicks made by 14 of their sighted participants and found large inter-individual differences in amplitudes and frequencies. Participants who produced intense clicks dominated by energy in the high-frequency part of the spectrum performed best in the echo detection task. This agrees with the point made above, that performance in experiments using self-generated sounds may benefit from specific training of how to produce efficient echo signals. Another point to be studied, is the use of the Echobot to test size discrimination. It is true that in our experiments, the object size was not modified, but different disk sizes could be used across sessions to measure size discrimination and detection thresholds. At the beginning of one session, the object can be replaced by another one fast. This could also include testing object of different materials to better study how different material properties affect size discrimination and detection threshold.

To summarize, the Echobot was developed to allow echolocation studies to combine the use of real sound-reflecting objects with psychophysical methods based on large sets of stimuli. The experiments illustrate the usefulness of the Echobot for obtaining individual threshold estimates through an adaptive psychophysical method, using both loudspeaker- and self-generated sounds, and for obtaining detailed acoustic measurements for a large set of distances from the reflecting disk. The Echobot has potential for testing many more parameters than those explored in the present experiments. We are currently testing the localization of sound reflections by placing two Echobots in front of the participant, whose task it is to determine whether the reflection came from the left or the right. Other potential applications could include varying the angle of the disk relative to the participant (in the present experiment only 0 or 90 degrees was tested), and manipulating the elevation of the disk. We may also explore differences in detection or localization of disks of different sizes and material. The Echobot is also a demonstrator of a training device that, once fully developed, may help newly blind people to get started with echolocation. The present experiments indicate that long training periods may be needed, and that training might benefit from using loudspeaker-generated sounds to specifically train the auditory system in addition to training the ability to produce efficient vocalizations.
